# Ag–NHC Complexes in the π-Activation of Alkynes

**DOI:** 10.3390/molecules28030950

**Published:** 2023-01-18

**Authors:** Shiyi Yang, Tongliang Zhou, Xiang Yu, Michal Szostak

**Affiliations:** Department of Chemistry, Rutgers University, 73 Warren Street, Newark, NJ 07102, USA

**Keywords:** silver, Ag, N-heterocyclic carbenes, NHCs, alkynes, p-functionalization, p-activation, Ag–NHCs, group 11 metals, gold–NHCs, copper–NHCs

## Abstract

Silver–NHC (NHC = N-heterocyclic carbene) complexes play a special role in the field of transition-metal complexes due to (1) their prominent biological activity, and (2) their critical role as transfer reagents for the synthesis of metal-NHC complexes by transmetalation. However, the application of silver–NHCs in catalysis is underdeveloped, particularly when compared to their group 11 counterparts, gold–NHCs (Au–NHC) and copper–NHCs (Cu–NHC). In this Special Issue on *Featured Reviews in Organometallic Chemistry*, we present a comprehensive overview of the application of silver–NHC complexes in the p-activation of alkynes. The functionalization of alkynes is one of the most important processes in chemistry, and it is at the bedrock of organic synthesis. Recent studies show the significant promise of silver–NHC complexes as unique and highly selective catalysts in this class of reactions. The review covers p-activation reactions catalyzed by Ag–NHCs since 2005 (the first example of p-activation in catalysis by Ag–NHCs) through December 2022. The review focuses on the structure of NHC ligands and p-functionalization methods, covering the following broadly defined topics: (1) intramolecular cyclizations; (2) CO_2_ fixation; and (3) hydrofunctionalization reactions. By discussing the role of Ag–NHC complexes in the p-functionalization of alkynes, the reader is provided with an overview of this important area of research and the role of Ag–NHCs to promote reactions that are beyond other group 11 metal–NHC complexes.

## 1. Introduction

After the first synthesis of a silver–NHC complex in 1993 by Arduengo [1], a breakthrough was achieved in 1998 by Lin et al., who found that Ag(I)–NHCs serve as efficient transfer reagents for the synthesis of other NHC–metal complexes [2]. Owing to the ease of synthesis of Ag–NHCs, their stability, and their avoidance of free carbenes, this reaction now represents one of the most straightforward approaches to the synthesis of metal–NHCs, which has significantly contributed to the widespread application of metal–NHC complexes in catalysis and biology [3,4,5,6,7,8,9,10,11,12,13,14,15,16,17,18].

Simultaneously, another major direction in the area of Ag–NHCs was their investigation as potential antimicrobial and anticancer agents, in which Ag–NHC complexes have been proposed to serve as slow releasing agents [19]. The area of silver–NHCs in medicinal chemistry is a vibrant area of research, with numerous reviews published in recent years [19,20,21,22,23,24].

Electronically, silver is a [Kr] 4d^10^s^1^ coinage metal [25,26,27,28], with the current market price [29] significantly lower than that of gold (Ag, USD 24.00, 1 oz, vs. Au, USD 1815, 1 oz), but higher than copper (Cu, USD 3.73, 1 oz), which is mirrored by the relative abundance of group 11 metals in the earth’s crust (Ag, 0.07 pm; Cu, 50 ppm; Au, 0.0011 ppm) [30]. The complexation of strongly s-donating NHC ligands to silver enhances the stability of silver, which can be exploited in catalysis [9,10,11,12,13,14,15,16,17,18,31,32,33,34,34,35,36,37]. Studies have found that compared with other group 11 metals, the Ag–NHC bond is longer (e.g., IPr–AgCl, 2.056 Å, Ag–Cl, 2.313 Å; IPr–AuCl, 1.941 Å, Au–Cl, 2.270 Å; IPr–CuCl, 1.881 Å, Cu–Cl, 2.106 Å), owing to the weaker p-donation of silver [38,39,40,41,42]. Furthermore, studies on the p-activation of alkynes [43,44,45,46,47,48,49,50,51] established that p to s metal donation, as well as metal to p* back-bonding, is in the order of Au > Cu > Ag [52,53]. Overall, these mechanistic studies highlight that (1) silver–NHC complexes are well-suited as transmetalating reagents, and (2) silver–NHC complexes are suitable for the electrophilic activation of alkynes by the p-coordination of cationic silver(I)–NHC to alkynes with electronic features complementary to other coinage metals.

In this Special Issue on *Featured Reviews in Organometallic Chemistry*, we present a comprehensive overview of the application of silver–NHC complexes in p-activation of alkynes. Several excellent reviews on silver and silver–NHC complexes have been published [31,32,33,34,34,35,36,37]. These reviews have addressed general aspects and applications of silver in organic synthesis [20,21,22,23,24,31,32,33,34,34,35,36,37]. A review specifically addressing the p-functionalization of alkynes by silver–NHC complexes has not been published thus far. The p-functionalization of alkynes [43,44,45,46,47,48,49,50,51] is one of the most important processes in chemistry, and it is used for the synthesis of a wide range of compounds in areas ranging from drug discovery, agrochemistry, biochemistry, and natural product synthesis to materials science [54,55,56,57,58]. Recent studies show significant promise for silver–NHC complexes as unique and highly selective catalysts in this class of reactions. The present review covers p-activation reactions catalyzed by Ag–NHCs since 2005 (the first example of p-activation in catalysis by Ag–NHCs) through December 2022. The review focuses on the structure of NHC ligands and p-functionalization methods. The review is divided into the following sections: (1) intramolecular cyclizations; (2) CO_2_ fixation; and (3) hydrofunctionalization reactions, where the relevant p-functionalization of olefins by silver–NHCs is also discussed for comparison purposes or to introduce the topic from a historical perspective. We hope that by discussing the role of Ag–NHC complexes in the p-functionalization of alkynes, the reader will be provided with an overview of this important area of research and the role of Ag–NHC to promote reactions that are beyond other group 11 metal–NHC complexes [25,26,27,28].

**Figure 1:**
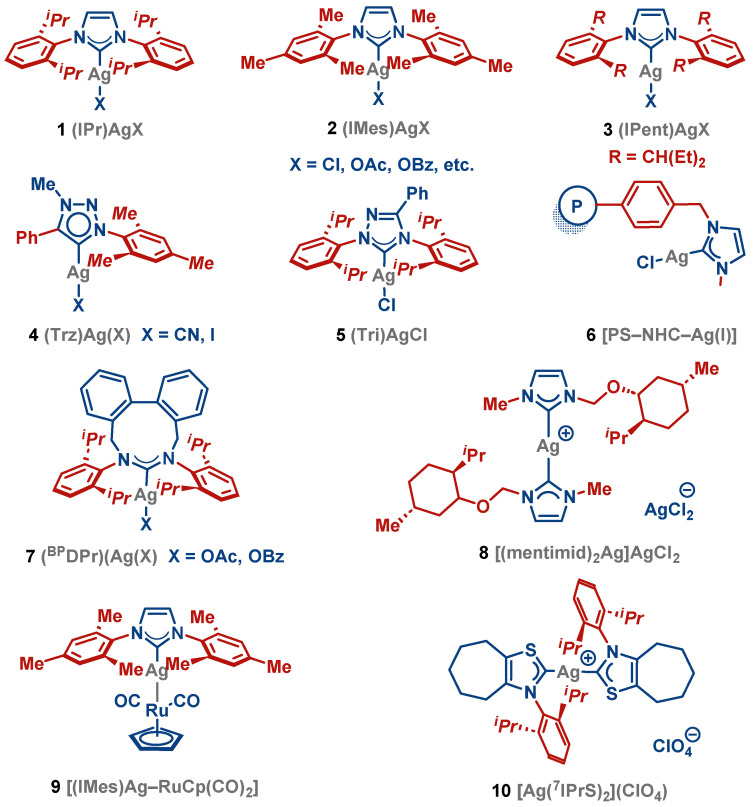
Structures of the most common Ag–NHC complexes used in π-functionalization of alkynes.

The structures of the most common Ag–NHC complexes used in the p-functionalization of alkynes are presented in Figure 1. Relevant bond lengths are presented in Table 1. For studies on the electronic and steric properties of NHC ligands, the reader is encouraged to consult the following reviews [3,4,5,6,7,8,9,10,11,12,13,14,15,16,17,18].

It should be noted that the Ag–C_(carbene)_ distances for the complexes shown in Figure 1 range between 2.056–2.091 Å for monomeric complexes and between 2.102–2.111 Å for bimetallic complexes. As expected, there is a much greater variation in the Ag–X bond lengths, with values ranging from 2.073–2.636 Å, for monomeric complexes and 2.617–2.952 Å for bimetallic complexes. These distances for the most common NHC–Ag complexes used in p-activation of alkynes are consistent with the major role of the counterion on their reactivity. Likewise, it should be noted that the three orbital contributions to the Ag–NHC bond involve s-donors d→p* (Ag to NHC p*-backdonation) and p→d (NHC to Ag p-donation). All three contributions should be considered in understanding the properties of Ag–NHC complexes in catalysis, which depend on the nature of the NHC scaffolds. The most effective Ag–NHC complexes discovered to date in the p-activation of alkynes are sterically hindered IPent, half-umbrella shaped thiazol-2-ylidene ^7^IPrS, and heteroatom-substituted 1,2,4-triazolylidene Tri NHC ligands. Future studies should carefully address the role of orbital contributions in elucidating the reactivity of Ag–NHCs in catalysis.

## 2. Intramolecular Cyclizations

**Scheme 1:**
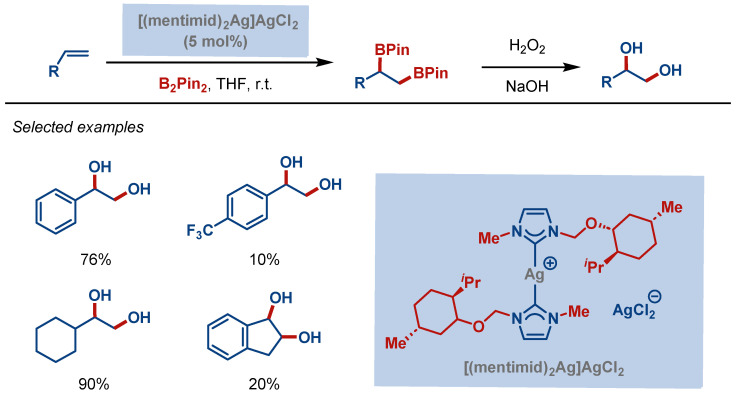
Ag(I)−NHC-catalyzed diboration of terminal and activated internal alkenes using B_2_Pin_2_, as reported by Fernandez and Peris [62].

**Scheme 2:**
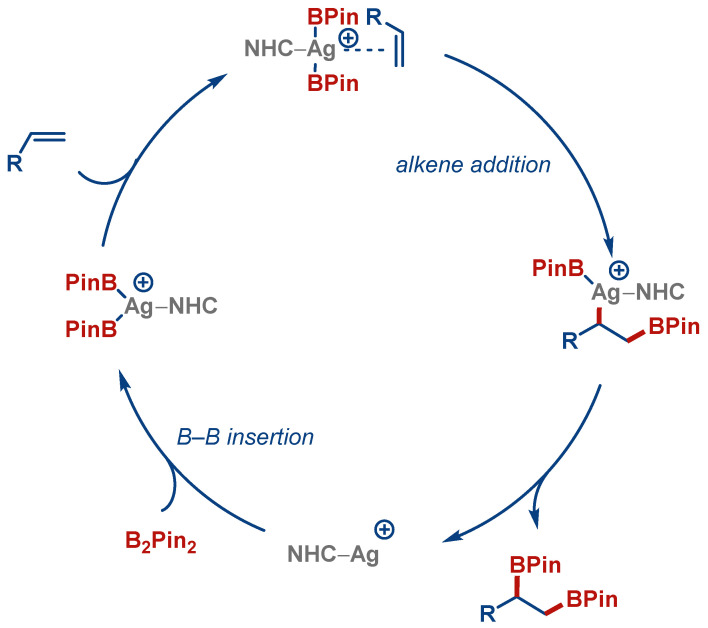
The catalytic cycle of Ag(I)−NHC-catalyzed diborylation of alkenes, as reported by Fernandez and Peris.

**Scheme 3:**
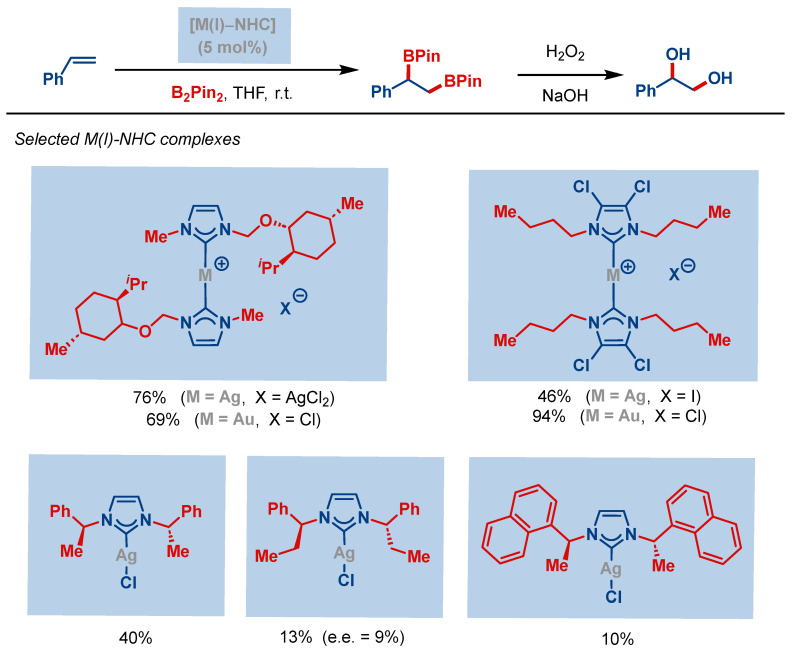
Metal−NHC-catalyzed diboration of internal alkenes using B_2_Pin_2_, as reported by Fernandez and Peris [65].

The first application of silver–NHC complexes in catalysis was reported by Fernandez and Peris in 2005 in the catalytic diboration of alkenes (Scheme 1) [62]. The authors developed a menthol-based bis-metallic Ag(I)–NHC complex, [(mentimid)_2_Ag]AgCl_2_, which provided relatively high reactivity in the diboration of terminal and activated internal alkenes using B_2_Pin_2_. Although no asymmetric induction was observed, the authors demonstrated the beneficial effect of Ag(I)–NHC in that the analogous Ag(I)-phosphine and cationic Ag^+^ salts were completely unreactive. The high reactivity was ascribed to the combination of the strong s-donation to break the B–B bond and the low propensity of Ag(I)–NHC in the b-hydride elimination of the alkyl–boryl intermediate due to low p-backbonding from Ag(I)–NHC. The proposed catalytic cycle is presented in Scheme 2. The key step involves the insertion of the Ag–NHC complexes into the B–B bond to yield diboryl species, which undergo alkene diborylation. An improved Ag(I)–NHC catalyst system was subsequently reported by the same authors (Scheme 3) [65]. This 2005 study set the stage for the exploration of Ag(I)–NHCs as efficient catalysts for the electrophilic activation of alkynes.

**Scheme 4:**
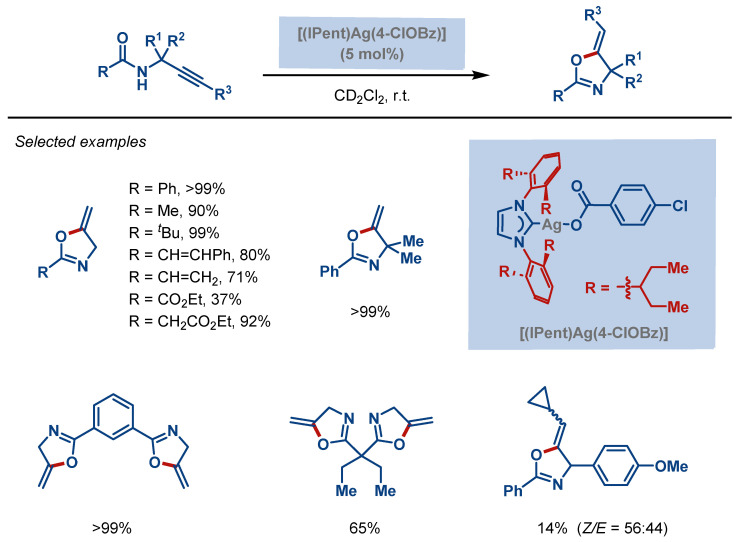
Ag(I)−NHC-catalyzed intramolecular cyclization of propargylic amides to yield oxazolidines, as reported by Hii, Nolan et al. [59].

In 2016, Hii, Nolan et al. reported an impressive study on the effect of Ag(I)–NHC carboxylates, [(NHC)Ag(O_2_CR)], in the intramolecular cyclization of propargylic amides to yield oxazolidines (Scheme 4) [59]. The authors synthesized a series of [(NHC)Ag(O_2_CR)] complexes with the goal of tuning electronic and steric properties of Ag(I)–NHC complexes by the NHC ligand, and achieving their stability by the carboxylate ligand. The balance between the stability and activity of Ag(I)–NHCs is a major consideration in catalysis. The authors identified [(IPent)Ag(4-ClOBz)], bearing a bulky IPent ligand and an electron-deficient 4-Cl-OBz throw-away ligand, as the most effective combination for catalysis. A range of propargylic amides was cyclized to oxazolidines under very mild room temperature conditions at 5 mol% catalyst loading. The substrate scope of this intramolecular cyclization was found to be complementary to Au–NHC catalysis [66,67,68], showcasing the synthetic utility of Ag(I)–NHC complexes in catalysis. An important finding of this study is the capacity to independently tune the sterics of the NHC ligand along with stability of the Ag(I)–NHC complex by the carboxylate ancillary ligand.

**Scheme 5:**
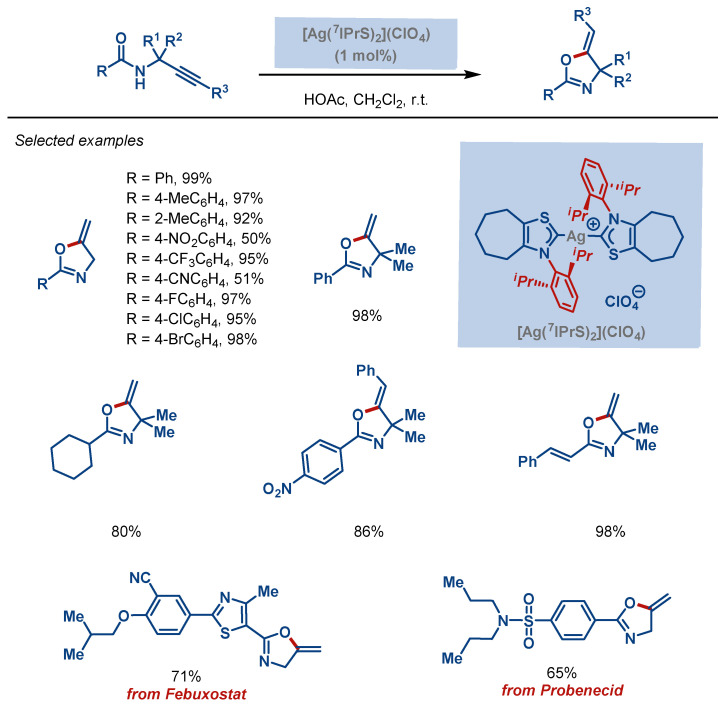
Ag(I)−NHC-catalyzed intramolecular cyclization of propargylic amides to afford oxazolidines, as reported by Zhang, Fang, Szostak et al. [64].

In 2022, we reported Ag(I)–thiazol-2-ylidene complexes and their application in the intramolecular cyclization to yield oxazolidines (Scheme 5) [64]. The non-classical framework of thiazol-2-ylidene offers new opportunities in catalysis due to its differentiated half-umbrella-shaped ligand structure and enhanced p-electrophilicity [69]. We found that these Ag(I)–thiazol-2-ylidene complexes are highly active in the electrophilic cyclization of propargylic amides. These reactions proceeded with excellent yields at room temperature in the presence of 1 mol% of the bis-NHC–Ag(I) complex. The most reactive was [Ag(^7^IPrS)_2_](ClO_4_), bearing a cycloheptyl thiazol-2-ylidene and perchlorate anion. The reaction was applied to the late-stage functionalization of pharmaceuticals, showcasing the mild reaction conditions and potential applications in medicinal chemistry.

**Scheme 6:**
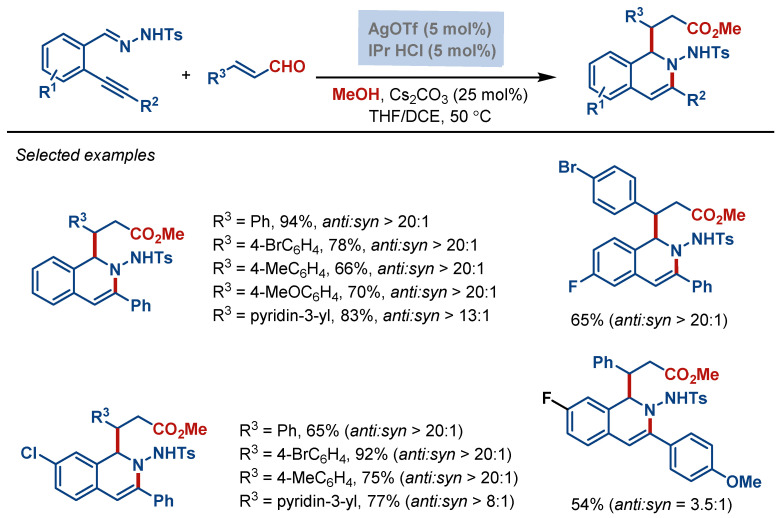
Ag(I)−NHC-catalyzed tandem tricomponent cyclization to afford 2-Amino-1,2-Dihydroisoquinolines, as reported by Wu et al. [70].

Another intramolecular cyclization involving Ag(I)–NHCs was reported by Wu et al. in 2010 (Scheme 6) [70]. In this work, the authors found that the combination of AgOTf and IPrHCl, in the presence of Cs_2_CO_3_ as a base, enabled a tandem tricomponent cyclization of N’-(2-alkynylbenzylidene)-hydrazides with a,b-unsaturated aldehydes and methanol to produce functionalized 1,2-dihydroisoquinolines. The reaction proceeds via the intramolecular cyclization of hydrazide onto the p-activated alkyne, followed by the nucleophilic addition of homoenolate and methanol. The scope of the synthesis of the 2-amino-1,2-dihydroisoquinoline products is significant, permitting for the synthesis of medicinally-relevant heterocycles.

**Scheme 7:**
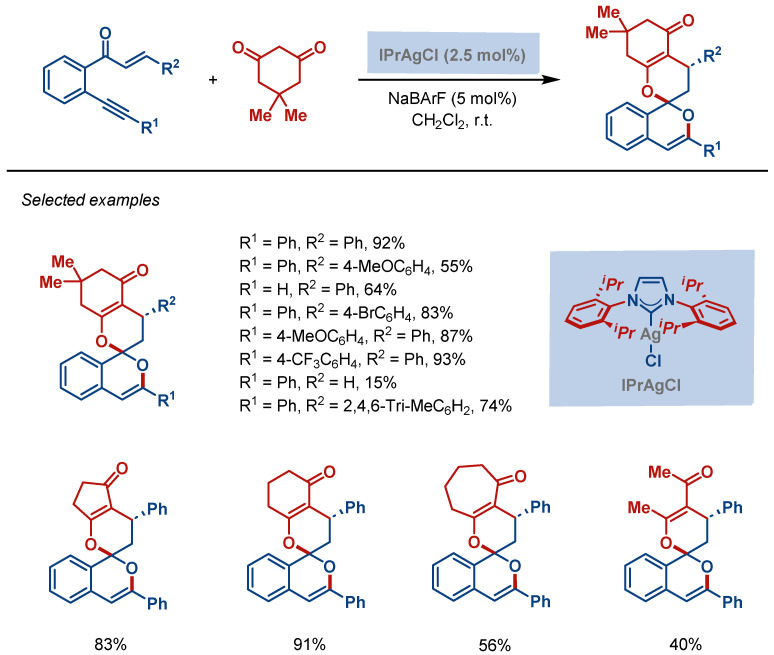
Ag(I)−NHC-catalyzed intramolecular 6-*endo*-*dig* cyclization to form 6-membered benzo-fused spirocycles, as reported by Hashmi et al. [71].

In 2019, Hashmi et al. reported a related approach based on Ag(I)–NHC-catalyzed intramolecular 6-*endo*-*dig* cyclization to form 6-membered benzo-fused spirocycles (Scheme 7) [71].

Mechanistically, the key step involves the intramolecular cyclization of the carbonyl group onto p-activated alkyne, followed by the intermolecular Michael addition and spirocyclization. The role of Ag–NHC is two-fold, acting as both an alkyne and enol p-activator to facilitate intramolecular cyclizations. The authors screened various group 11 metal catalysts for this intriguing spriocyclization and found that [(IPr)AgCl]/NaBAr^F^ is the most effective catalyst. This system outperformed simple Ag salts, such as AgNTf_2_, AgBF_4_, or AgOTf, as well as various Au and Cu catalysts, such as AuBr_3_, [(IPr)AuCl_3_], or [(IPr)CuCl]/NaBAr^F^. The methodology is particularly notable for its broad substrate scope and rapid, convergent approach to biologically privileged 6,6-spiroketals, highlighting the utility of Ag(I)–NHCs catalysis in the synthesis of *O*-heterocycles.

**Scheme 8:**
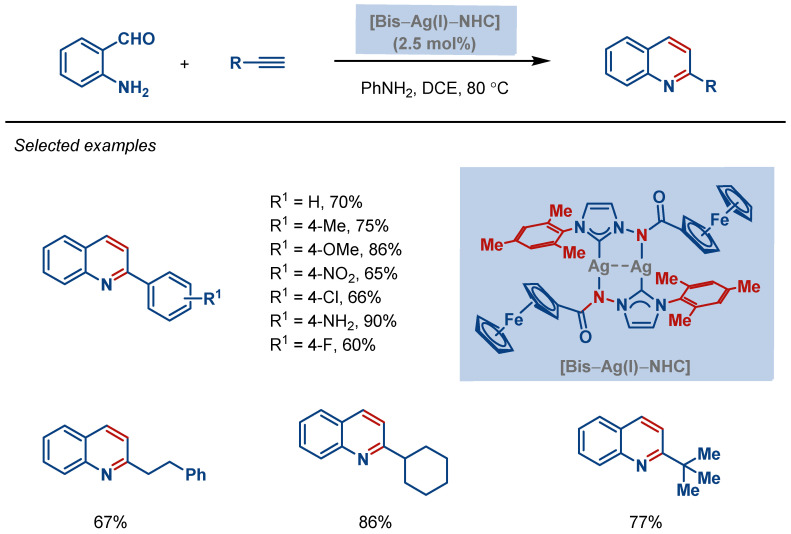
Bimetallic Ag(I)−NHC-catalyzed tandem tricomponent cyclization to afford 2-functionalized quinolines, as reported by Bera et al. [72].

In 2013, Bera et al. reported an interesting synthetic approach to quinolines by Ag(I)–NHC catalysis (Scheme 8) [72]. In contrast to the approaches described in Scheme 3, Scheme 4, Scheme 5 and Scheme 6, this reaction involves Ag(I)–NHC-catalyzed alkyne hydroamination, followed by condensation with 2-aminobenzaldehyde. The catalyst used in this case is a bimetallic Ag(I)–NHC bridged by two anionic N-Mes/N-ferrocenoyl amide ligands. Although no information was provided on the comparative activity of other complexes, the scope of the method appears to be quite broad. The reaction delivers important 2-funtionalized quinoline heterocycles in high yields via a three-component coupling.

**Scheme 9:**
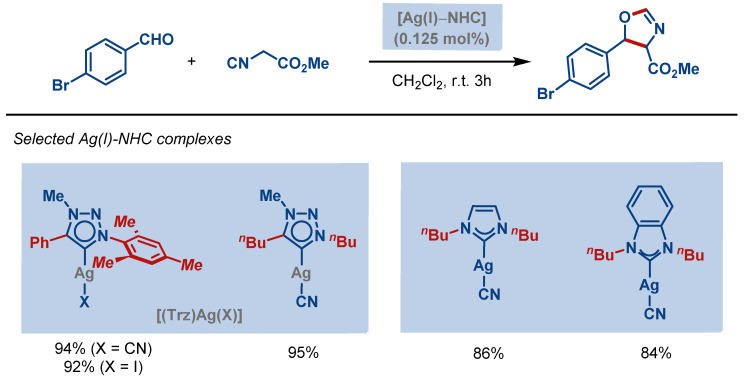
Ag(I)−NHC-catalyzed synthesis of oxazolines from benzaldehydes and isocyanates, as reported by Albrecht et al. [60].

In light of the reactions described above, it is important to mention the Ag(I)–NHC-catalyzed synthesis of oxazolines from benzaldehydes and isocyanates, as reported by Albrecht in 2015 (Scheme 9) [60]. This reaction features non-classical silver triazolylidene complexes readily prepared by the Lin method from Ag_2_O and the corresponding triazolium salts. In the complex synthesis, the use of CH_3_CN as a solvent resulted in C–C bond activation and the formation of [(trz)Ag(CN)] complexes, while [(trz)Ag(X)] complexes were formed in CH_2_Cl_2_. These Ag(I)–trz complexes showed slightly higher reactivity than the analogous imidazol-2-ylidene Ag(I) complexes in the synthesis of oxazolines. The reaction was highly effective, even at 0.10 mol% catalyst loading, showcasing the powerful role of Ag(I)–NHC in promoting intramolecular cyclizations.

## 3. CO_2_ Fixation

**Scheme 10:**
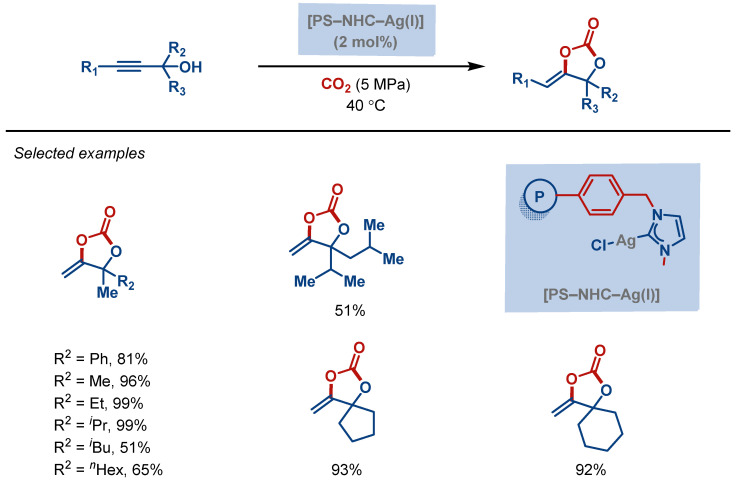
Polystyrene-supported Ag(I)−NHC-catalyzed CO_2_ fixation into propargylic alcohols, as reported by Jiang et al. [75].

The ability to incorporate CO_2_ in carbon–carbon bond-forming reactions as a renewable C1 synthon is of great interest in organic synthesis [73,74]. In 2013, Jiang et al. reported polystyrene supported Ag(I)–NHC complexes, [PS–NHC–Ag(I)], for CO_2_ fixation into propargylic alcohols (Scheme 10) [75]. The [PS–NHC–Ag(I)] complexes were readily prepared using appropriately substituted N-alkyl-imidazoles with polystyrene-supported benzyl chloride. The most active was the N-Me substituted complex. Interestingly, the analogous Cu–NHC complex, [PS–NHC–Cu(I)], showed no activity under the reaction conditions. These [PS–NHC–Ag(I)] complexes promoted the carboxylative cyclization of a range of propargylic alcohols to terminal alkylidene cyclic carbonates in generally excellent yields under 5 MPa pressure of CO_2_ at 40 °C. This approach by Jiang et al. has several benefits, including high catalytic activity, ease of catalyst separation, and catalyst recyclability.

**Scheme 11:**
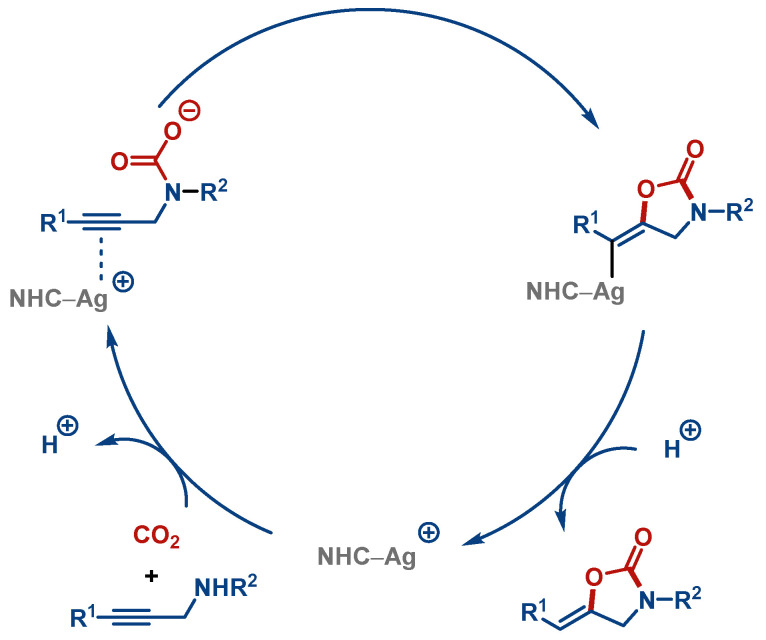
The general catalytic cycle of Ag(I)−NHC-catalyzed CO_2_ fixation to propargylamines.

The proposed catalytic cycle is presented in Scheme 11. The reaction involves the p-activation of the alkyne by the cationic Ag–NHC species, followed by the nucleophilic attack of the carbamate anion. Protonolysis regenerates the active Ag–NHC species. This represents a general mechanism for cyclization reactions mediated by Ag–NHC complexes.

**Scheme 12:**
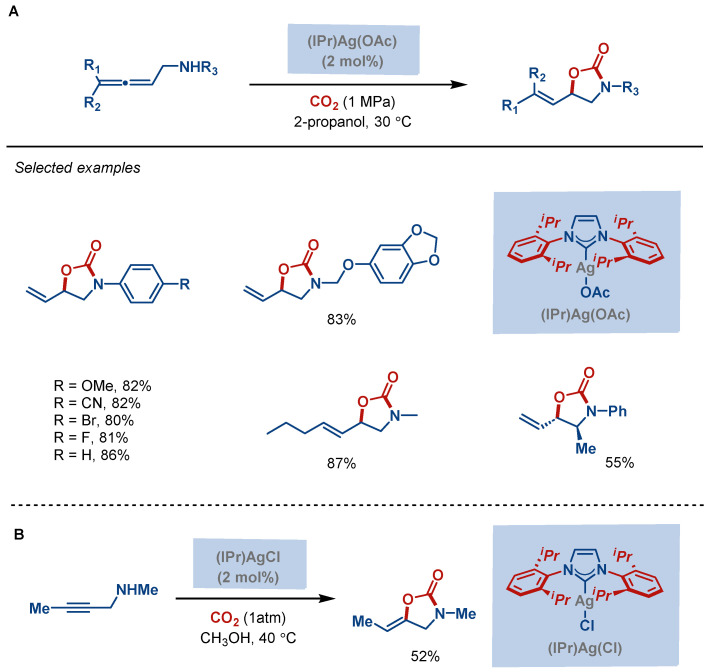
(**A**) Ag(I)−NHC-catalyzed CO_2_ fixation into allenylmethylamines, and (**B**) into propargylamines, as reported by Ikariya et al. [76].

In 2015, Ikariya et al. reported the Ag(I)–NHC-catalyzed fixation of CO_2_ into allenylmethylamines (Scheme 12A) [76]. The authors identified [(IPr)Ag(OAc)] as the most effective catalyst to afford alkenyl-1,3-oxazolidin-2-ones. The choice of metal, ancillary ligand, and counterion was critical for this process. The analogous Au and Cu complexes, [(IPr)Au(OAc)] and [(IPr)Cu(OAc)], were completely ineffective, while [(IPr)Ag(Cl)] showed minimal activity (<10%). The reaction showed a good scope of allenylmethylamines at an atmospheric pressure of CO_2_ at 30 °C. Mechanistically, two competing pathways were proposed, carboxylative cyclization leading to alkenyl-1,3-oxazolidin-2-ones and intramolecular hydroamination resulting in 2,-5-dihydropyrroles, initiated by p-coordination to the internal or external allene double bond. Interestingly, the same group reported intramolecular carboxylative cyclization of propargylamines to alkylidene-1,3-oxazolidin-2-ones, mediated by [(IPr)AgCl], in modest yields (Scheme 12B).

**Scheme 13:**
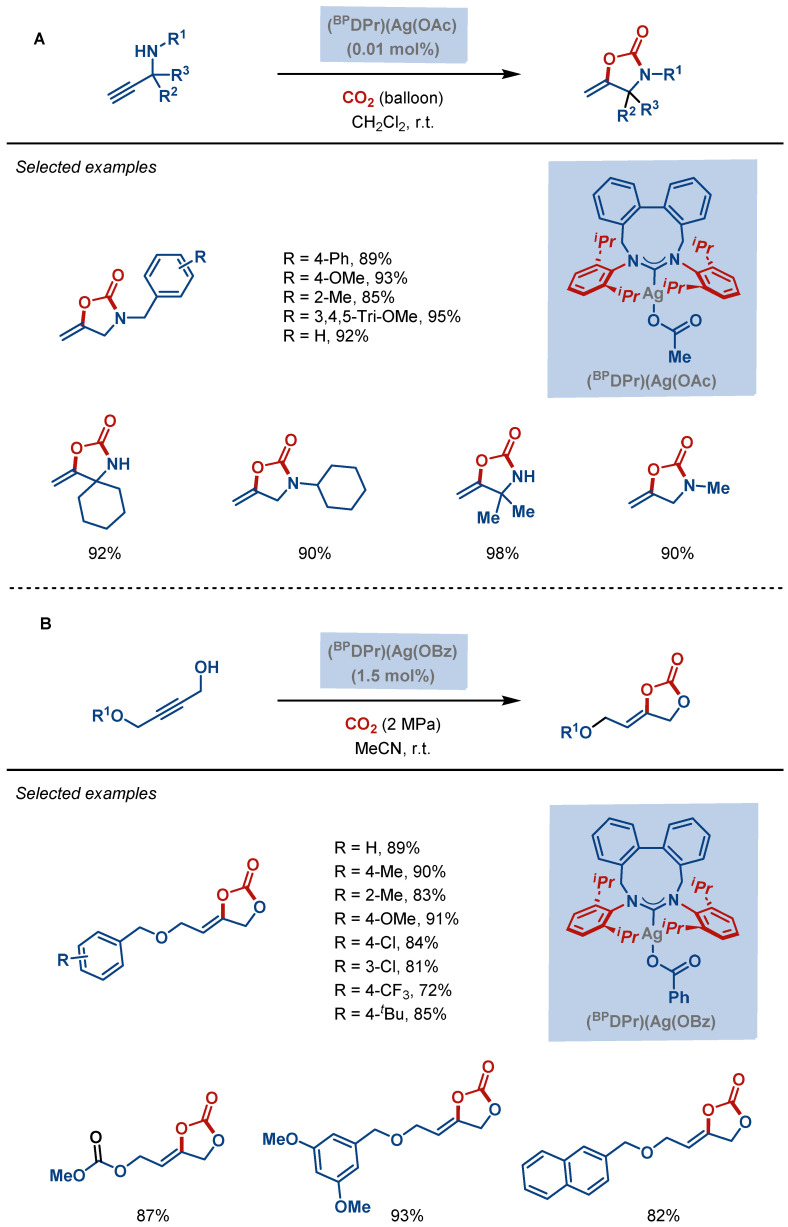
(**A**) Ag(I)−NHC-catalyzed carboxylative cyclization of propargylic alcohols, and (**B**) amines, as reported by Cervantes-Reyes, Hashmi et al. [61].

In 2021, an important breakthrough was reported by Cervantes-Reyes, Hashmi et al. in identifying ring-expanded Ag(I)–NHC complexes as efficient catalysts for the carboxylative cyclization of propargylic alcohols and amines (Scheme 13) [61]. The most active complexes were [(^BP^DPr)(Ag(OAc)] and [(^BP^DPr)(Ag(OBz)], featuring a nine-membered, bulky NHC ligand (^BP^DPr = 1,3-bis(2,6-diisopropylphenyl)-1,3-diazonine-2-ylidene) and carboxylate counterions. These complexes are characterized by some of the largest buried volumes reported for [(NHC)AgX] complexes to date, ([(^BP^DPr)(Ag(OAc)]: %V*_bur_* = 52.9%; [(^BP^DPr)(Ag(OBz)]: %V*_bur_* = 54.5%). The scope of the carboxylative cyclization mediated by these ring-expanded NHCs is particularly broad, which has been ascribed to the steric distribution of the ligand on the metal center. The highlight is the ability to promote the carboxylative cyclization of unsubstituted propargylic alcohols and amines to afford terminal and internal unsubstituted oxazolidinones and cyclic a-methylene carbonates in excellent yields.

## 4. Hydrofunctionalization Reactions

**Scheme 14:**
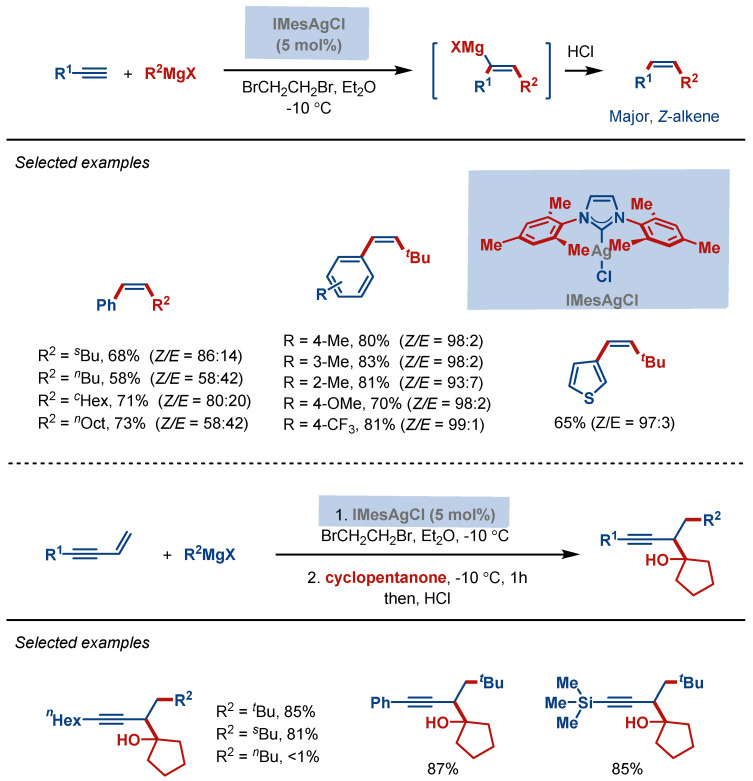
Ag(I)–NHC-catalyzed carbomagnesiation of alkynes, as reported by Kambe et al. [80].

The catalytic hydrofunctionalization of alkynes is among the most useful transformations in organic synthesis [77,78,79]. In 2009, Kambe et al. reported the carbomagnesiation of alkynes catalyzed by Ag(I)–NHC complexes (Scheme 14) [80]. This reaction proceeds in the presence of [(IMes)AgCl] as a catalyst, alkyl Grignard reagent as a nucleophile, and BrCH_2_Br as a stoichiometric additive. Ag–NHCs are the preferred catalysts over simple silver salts, such as AgOTs, and Ag–phosphine systems, such as AgOTs/PPh_3_, affording higher yields and *Z*:*E* selectivity up to 99:1. Mechanistically, the reaction involves the formation of an alkyl silver complex, followed by anti-alkyne insertion and transmetalation. The scope of this process is broad with respect to aryl alkynes using *t*-BuMgCl as a nucleophile. However, lower selectivity was observed with less sterically hindered Grignard reagents. The authors extended the utility of this process to the carbofunctionalization of enynes and trapping with carbonyl electrophiles.

**Scheme 15:**
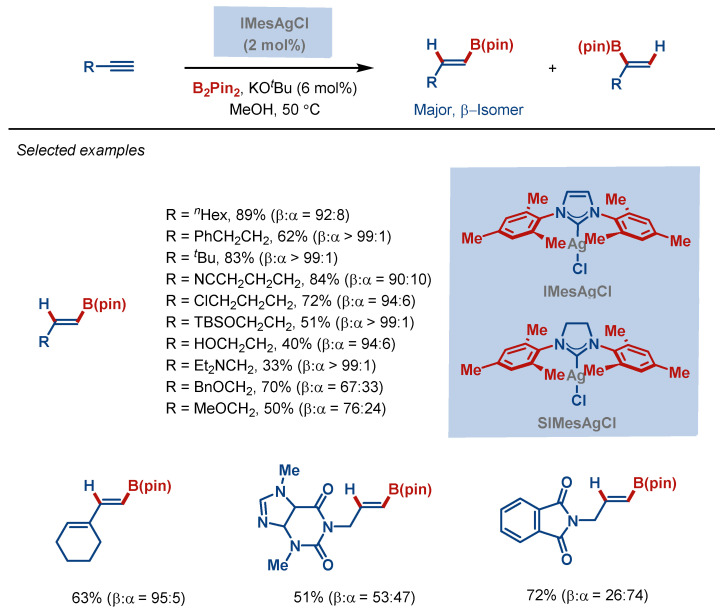
Ag(I)−NHC-catalyzed hydroboration of alkynes, as reported by Yoshida et al. [81].

In 2014, a significant method for the hydroboration of alkynes catalyzed by Ag(I)–NHC complexes was reported by Yoshida et al. (Scheme 15) [81]. [(IMes)AgCl], in the presence of catalytic KO*t*Bu and B_2_Pin_2_ (1 equiv) in MeOH at 50 °C, was identified as the optimal system for this transformation. Interestingly, the imidazolin-2-ylidene analogue, [(SIMes)AgCl], showed almost identical reactivity, while the imidazol-2-ylidene counterpart, [(IPr)AgCl], was completely unreactive under the tested conditions. The scope of the reaction is very broad and involves terminal aliphatic alkynes and internal aromatic alkynes. The yields and selectivity for the formation of b-hydroboration products are generally high to excellent. Mechanistically, the key step is the formation of [(IMes)Ag–BPin] species by σ-metathesis between [(IMes)Ag–O*t*Bu] and B_2_Pin_2_. This [(IMes)Ag–BPin] species increases across the alkyne bond to generate β-boryl–organosilver, which undergoes protonolysis.

**Scheme 16:**
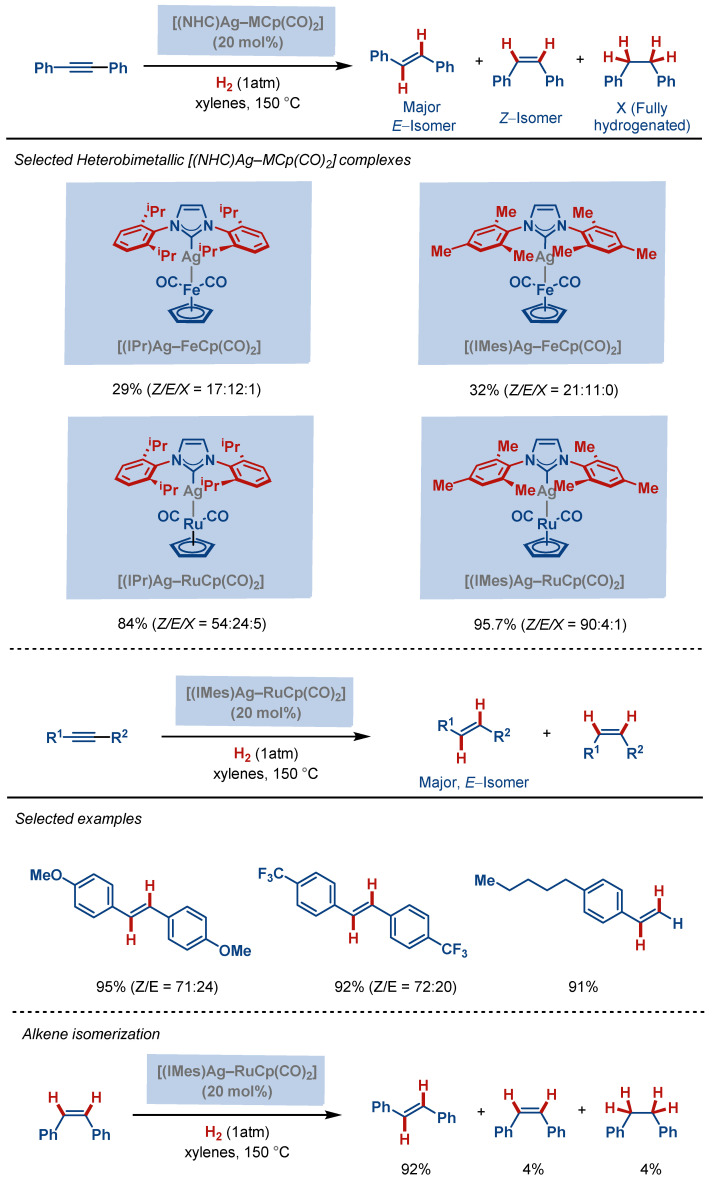
Bimetallic [Ru-Ag(I)−NHC]-catalyzed *E*-selective hydrogenation of alkynes, as reported by Mankad et al. [63].

In 2014, Mankad et al. reported an intriguing *E*-selective hydrogenation of alkynes by [Ag–Ru] bimetallic catalysis (Scheme 16) [63]. The most effective catalyst system is [(IMes)Ag–RuCp(CO)_2_] under atmospheric pressure of H_2_ in xylenes at 150 °C. The bimetallic cooperation is critical to this process, as no reaction is observed for any of the catalyst systems alone. IPr and Cu, as well as FeCp(CO)_2_, can be used; however, the yields and selectivity are lower than with the [(IMes)Ag–RuCp(CO)_2_] complex. Mechanistically, bimetallic H_2_ activation is followed by *syn*-alkyne insertion into [(IMes)–Ag–H] to afford a-alkenyl–Ag(NHC) intermediate and protonolysis by [RuCp(CO_2_)–H]. The authors demonstrated that *Z*/*E* alkene isomerization takes place under the reaction conditions. The functional group tolerance of this method is broad, as demonstrated by the Glorius robustness test, where only aldehydes were found to inhibit the reaction rate. This study demonstrates the potential of Ag–NHCs as an effective class of ligands in the emerging area of bimetallic catalysis [82].

**Scheme 17:**
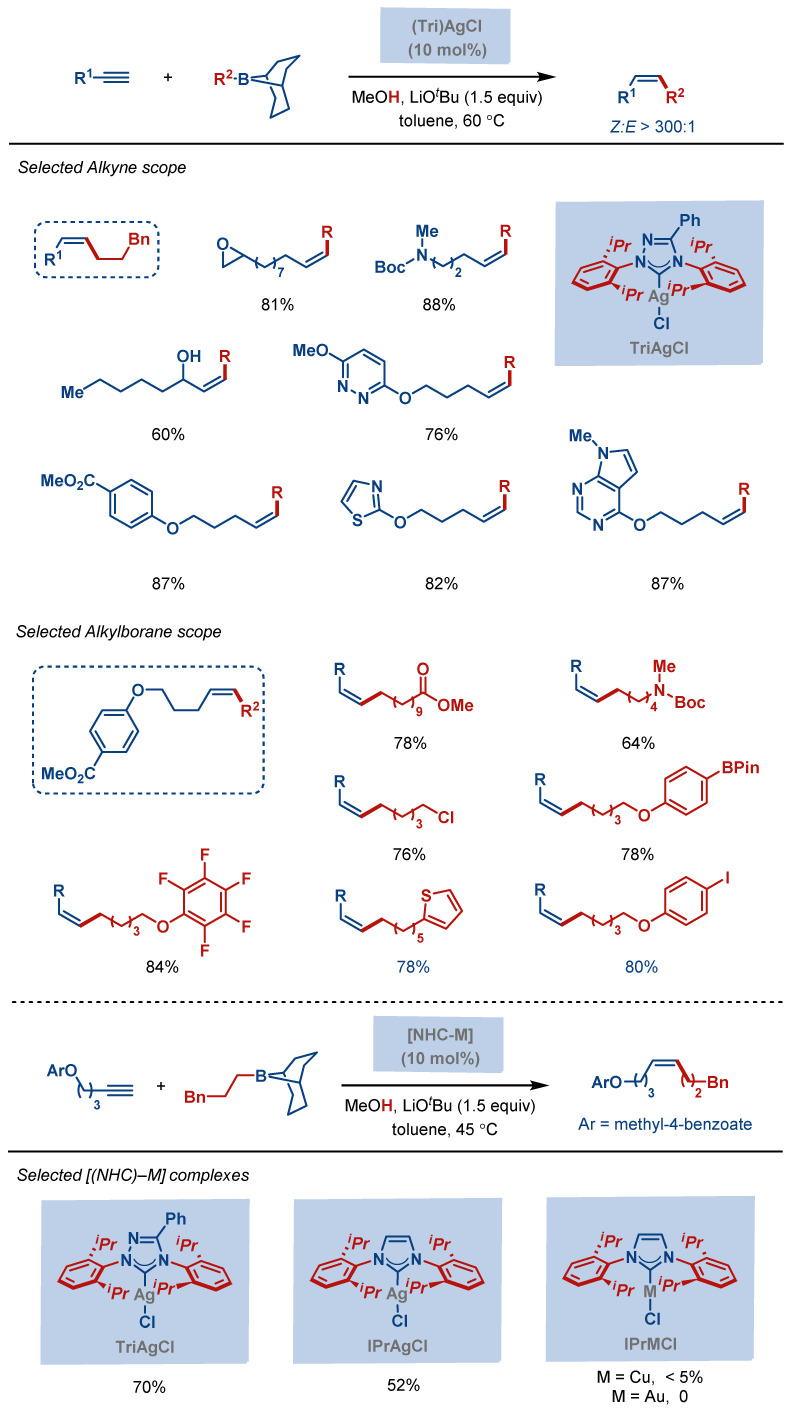
Ag(I)−NHC-catalyzed hydroalkylation of alkynes, as reported by Lalic et al. [83].

In 2019, the Lalic group reported the hydroalkylation of alkynes catalyzed by Ag(I)–NHC complexes (Scheme 17) [83]. The most intriguing feature of this report is the use of 1,2,4-triazolylidene NHC, [(Tri)AgCl] (Tri = 1-phenyl-2,4-Dipp-1,2,4-triazolylidene) as a more effective ligand than the classical imidazol-2-ylidene, [(IPr)AgCl]. Furthermore, the analogous Cu complex, [(IPr)CuCl], was completely unreactive. 1,2,4-Triazol-5-ylidenes are significantly less basic than imidazol-2-ylidenes (p*K*_a_ = 16.1 vs. 21.5, calculated values, DMSO) [84], which may contribute to the higher reactivity of [(Tri)AgCl] vs. [(IPr)AgCl]. The reaction yields *Z*-alkenes with full stereoselectivity and excellent functional group tolerance.

**Scheme 18:**
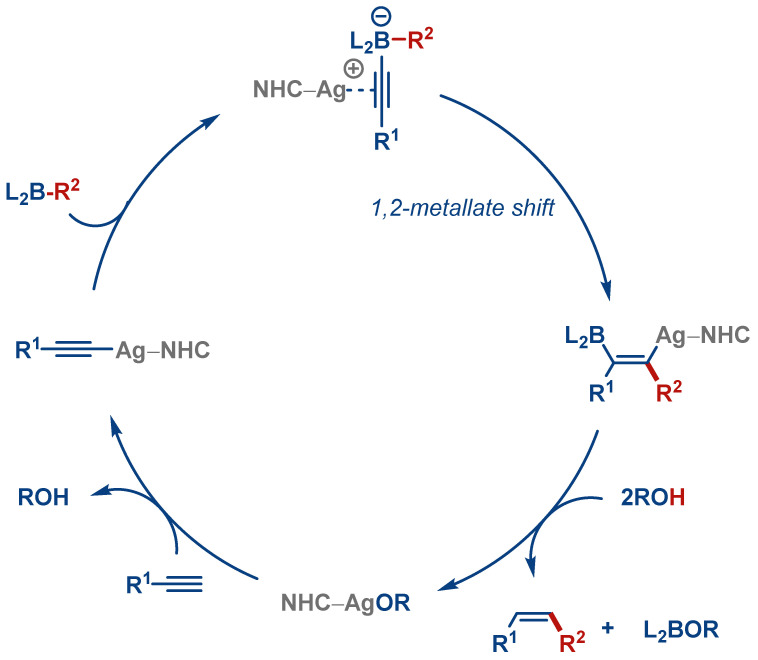
The catalytic cycle of Ag(I)−NHC-catalyzed hydroalkylation of alkynes, as reported by Lalic et al. [83].

Mechanistically, the reaction involves the combination of the s- and p-activation of alkynes, which may further explain the superior reactivity of Ag–NHCs vs. Cu–NHCs. The authors proposed that silver acetylide reacts with alkyl borane, followed by a 1,2-metalate shift after p-activation. The catalytic cycle is completed by protodemetalation and protodeboronation (Scheme 18). This report provides a clear example of the advantages of using Ag(I)–NHCs in catalysis by combining two activation modes inherently favored by silver.

## 5. Conclusions and Outlook

In summary, over the past 15 years, significant advances have been made regarding the use of Ag–NHC complexes for the synthetically important functionalization of alkynes. Among the major advantages of Ag–NHCs is the enhanced stability of silver rendered possible by the strongly s-donating NHC ligands, ligand amplified reactivity in several general classes of reactions, and the improved reactivity over other group 11 metals. In particular, the progress has been considerable in the following generic classes of reactions: intramolecular cyclizations, CO_2_ fixation, and hydrofunctionalization reactions. These reactions provide heterocyclic products important for medicinal chemistry research and functionalized building blocks for organic synthesis. Among the reported reactions, the most noteworthy are processes that specifically demonstrate the beneficial role of Ag–NHCs, such as the electrophilic cyclization of propargylic amides, CO_2_ fixation, the bimetallic Ag–Ru hydrogenation of alkynes, and the hydroalkylation of alkynes.

**Figure 2:**
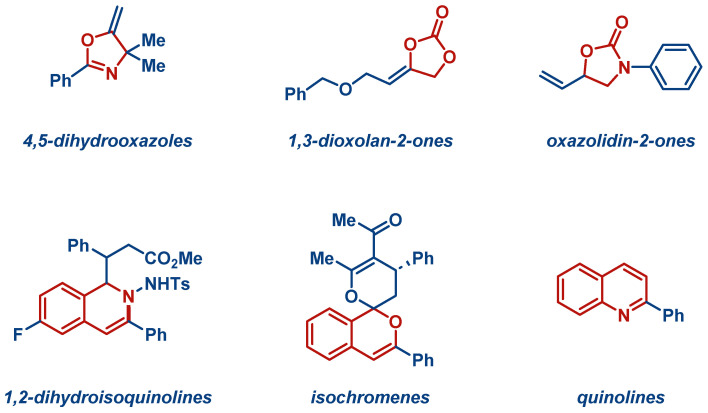
Heterocyclic scaffolds (4,5-dihydrooxazoles [59,64,85]; 1,3-dioxolan-2-ones [61,86]; oxazolidin-2-ones [76,87]; 1,2-dihydroisoquinolines [70,88]; isochromenes [71,89]; quinolines [72,90]; synthesized by Ag(I)−NHC complexes.

An interesting consideration is the fact that Ag–NHC complexes appear to be particularly well-suited for the synthesis of heterocycles, which play a role in potential therapeutic agents (Figure 2) [85,86,87,88,89,90]. This reactivity of Ag–NHCs in the p-activation of alkynes bodes well for the broad practical application of this class of M–NHCs in medicinal chemistry research.

Despite significant progress, there are several areas that should be addressed in the future to render this Ag–NHC manifold of even more general utility in organic synthesis: (1) mechanistic studies are urgently needed to elucidate the role of Ag–NHCs in comparison with other group 11 metal–NHC complexes; (2) the role of NHC ligands has rarely been explored in Ag–NHC catalysis, with majority of reactions limited to testing only IPr and IMes ligands; (3) although the mechanistic basis for several alkyne functionalization manifolds using Ag–NHCs has been established, few reactions have been explored using this catalysis manifold; (4) the role of the counterion has not been fully elucidated, with simple carboxylate anions typically preferred for the activation of alkynes; and (5) the development of asymmetric processes using chiral Ag–NHCs has not yet been accomplished.

In the group of coinage metals, silver has several major advantages over Au and Cu, including low price and wide availability (vs. Au), the capacity to promote various activation modes, ease of transmetalation, and complementary electronic properties, such as the p to s metal donation and the metal to p* backbonding. The reported studies clearly demonstrate that researchers using group 11 metal–NHC complexes should always consider Ag(I)–NHCs in the development of catalytic processes.

## Figures and Tables

**Table 1:** Ag–C_(carbene)_ bond lengths of the most common Ag–NHC complexes used in the π-functionalization of alkynes. Ag–X bond lengths are shown for comparison.

Entry	Complex No.	[(NHC)Ag(X)]	Ag–C Bond Length (Å)	Ag–X Bond Length (Å)	Reference
1	**1**	[(IPr)Ag(Cl)]	2.056	2.313 (X = Cl)	[41]
2	**2**	[(IMes)Ag(Cl)]	2.056	2.314 (X = Cl)	[42]
3	**3a**	[(IPent)Ag(OAc)]	2.067	2.111 (X = OAc)	[59]
4	**3b**	[(IPent)Ag(OBz)]	2.059	2.100 (X = OBz)	[59]
5	**3c**	[(IPent)Ag(4-ClOBz)]	2.064	2.100 (X = 4-ClOBz)	[59]
6	**4a**	[(Trz)Ag(CN)]	2.087	2.073 (X = CN)	[60]
7	**4b**	[(Trz)Ag(I)]	2.091	2.636 (X = I)	[60]
8	**7a**	[(^BP^DPr)Ag(OAc)]	2.089	2.112 (X = OAc)	[61]
9	**7b**	[(^BP^DPr)Ag(OBz)]	2.089	2.122 (X = OBz)	[61]
10	**8**	[(mentimid)_2_Ag(AgCl_2_)]	2.102	2.952 (X = AgCl_2_)	[62]
11	**9**	[(IMes)Ag(RuCp(CO)_2_)]	2.111	2.617 (X = RuCp(CO)_2_)	[63]
12	**10**	[Ag(^7^IPrS)_2_]^+^[ClO_4_]^−^	2.081	-	[64]

## Data Availability

Not applicable.
